# Corrigendum

**DOI:** 10.1002/rcr2.169

**Published:** 2016-06-13

We would like to draw the reader's attention to an error in the following article:

Murakami Y , Oki M , Saka H , Kajikawa S , Murakami A , and Ishida A . Disseminated cryptococcosis presenting as mediastinal and hilar lymphadenopathy in an immunocompetent patient. Respirol Case Rep. 2016; 4: e00167. 10.1002/rcr2.167PMC496985327512567

The affiliations of the authors were interchanged. The authors' affiliations should be listed as:

Yasushi Murakami^1^, Masahide Oki^1^, HideoSaka^1^, Shigehisa Kajikawa^2^, Ayuka Murakami^3^ & Akane Ishida^1^

^1^Department of Respiratory Medicine, Nagoya Medical Center, Nagoya, Japan.

^2^Department of Respiratory Medicine, Tokoname Municipal Hospital, Tokoname, Japan.

^3^Department of Neurology, Nagoya Medical Center, Nagoya, Japan.

The publisher apologises for this error and any confusion it may have caused.

